# The Dystonia Coalition: A Multicenter Network for Clinical and Translational Studies

**DOI:** 10.3389/fneur.2021.660909

**Published:** 2021-04-08

**Authors:** Gamze Kilic-Berkmen, Laura J. Wright, Joel S. Perlmutter, Cynthia Comella, Mark Hallett, Jan Teller, Sarah Pirio Richardson, David A. Peterson, Carlos Cruchaga, Codrin Lungu, H. A. Jinnah

**Affiliations:** ^1^Department of Neurology, Emory University School of Medicine, Atlanta, GA, United States; ^2^Department of Neurology, Washington University School of Medicine, St. Louis, MO, United States; ^3^Department of Neurology, Radiology, Neuroscience, Physical Therapy and Occupational Therapy, Washington University School of Medicine, St. Louis, MO, United States; ^4^Department of Neurological Sciences, Rush University Medical Center, Chicago, IL, United States; ^5^Human Motor Control Section, National Institute of Neurological Disorders and Stroke (NINDS), National Institute of Health (NIH), Bethesda, MD, United States; ^6^Dystonia Medical Research Foundation, Chicago, IL, United States; ^7^Department of Neurology, University of New Mexico Health Sciences Center, Albuquerque, NM, United States; ^8^Institute for Neural Computation, University of California, San Diego, La Jolla, CA, United States; ^9^Department of Psychiatry, Hope Center Program on Protein Aggregation and Neurodegeneration, Washington University School of Medicine, St. Louis, MO, United States; ^10^Division of Clinical Research, National Institute of Neurological Disorders and Stroke (NINDS), National Institute of Health (NIH), Bethesda, MD, United States; ^11^Department of Human Genetics, Emory University School of Medicine, Atlanta, GA, United States

**Keywords:** dystonia, blepharospasm, cervical dystonia, laryngeal dystonia, rare diseases, spasmodic dysphonia, torticollis, writer's cramp

## Abstract

Dystonia is a movement disorder characterized by sustained or intermittent muscle contractions causing abnormal postures, repetitive movements, or both. Research in dystonia has been challenged by several factors. First, dystonia is uncommon. Dystonia is not a single disorder but a family of heterogenous disorders with varied clinical manifestations and different causes. The different subtypes may be seen by providers in different clinical specialties including neurology, ophthalmology, otolaryngology, and others. These issues have made it difficult for any single center to recruit large numbers of subjects with specific types of dystonia for research studies in a timely manner. The Dystonia Coalition is a consortium of investigators that was established to address these challenges. Since 2009, the Dystonia Coalition has encouraged collaboration by engaging 56 sites across North America, Europe, Asia, and Australia. Its emphasis on collaboration has facilitated establishment of international consensus for the definition and classification of all dystonias, diagnostic criteria for specific subtypes of dystonia, standardized evaluation strategies, development of clinimetrically sound measurement tools, and large multicenter studies that document the phenotypic heterogeneity and evolution of specific types of dystonia.

## Introduction to Dystonia

Dystonia is a movement disorder characterized by sustained or intermittent muscle contractions causing abnormal postures, repetitive movements, or both (1). Dystonic movements are typically patterned, twisting, or may resemble tremor. Dystonia is often initiated or worsened by voluntary action and associated with overflow muscle activation. Dystonia is not a single disorder but a family of heterogenous disorders with varied clinical manifestations and many different causes (2, 3).

The many different clinical manifestations of dystonia are grouped according to age at onset, body region affected, temporal aspects, and associated clinical features (1). The term “isolated dystonia” (previously known as “primary dystonia”) is used when dystonia is the only movement disorder identified, with or without tremor. In contrast, the term “combined dystonia” (previously known as “secondary dystonia” or “dystonia-plus”) is used when dystonia is combined with other neurological problems, such as parkinsonism, myoclonus, or ataxia). The most common subtypes of isolated dystonia emerge in adults over a period of weeks or months in one region of the body, with spread to other regions over many years. Any region of the body can be affected, but the most common regions include the neck (cervical dystonia, also known as torticollis), the face (blepharospasm and related craniofacial dystonias, sometimes called Meige syndrome), the larynx (laryngeal dystonia, also known as spasmodic dysphonia), or a limb (e.g., writer's cramp or musician's dystonia). Children are less commonly affected than adults, although more likely to advance to more severe generalized forms.

There are many known etiologies for dystonia. They include lesions of the nervous system, exposure to drugs or medications, infections and autoimmune processes, and other causes (2–4). However, for the vast majority of cases of isolated dystonia, a cause cannot be identified, even after extensive laboratory testing. Approximately 10–15% of cases have an affected family member. This observation points to inherited mechanisms. More than 100 genes capable of causing dystonia are known, most of which cause early-onset or combined forms of dystonia (2, 4, 5). Recent whole-exome sequencing studies have suggested that an etiology can be identified in ~20% of cases, depending on the associated clinical features (6, 7). A genetic etiology is disclosed more often in young-onset cases, those where dystonia is combined with other problems, or those with a family history of dystonia. A genetic etiology is found in only ~4% of the most common adult-onset cases.

Dystonia causes substantial disability (8, 9). For example, cervical dystonia is associated with neck muscle spasms that make it difficult for patients to control head movements for basic activities of daily living such as looking straight ahead to drive a car, read, see a computer or television screen, or even walk. Blepharospasm is associated with periocular spasms leading to frequent sustained eye closures. These spasms make it difficult to do many of same activities of daily living and may render subjects functionally blind. Laryngeal dystonia is associated with spasms of laryngeal muscles making it difficult to speak and communicate with others. Patients with limb dystonia may have trouble writing, typing, or walking. When this affects professionals such as musicians, dystonia can end a career. Patients with broader distributions of dystonia such as segmental or generalized patterns have even greater disability.

In addition to the abnormal movements that interfere with activities of daily living, dystonia is often associated with pain. Approximately two thirds of all patients with cervical dystonia have significant pain in the neck or shoulders (10). Approximately half of all patients with dystonia of the upper limb have arm or hand pain (11, 12). Many patients with generalized dystonia have pain relating to the most prominent areas of spasm. In addition to muscle pain, orthopedic complications that result from abnormal postures are a source of chronic pain for many patients with dystonia.

These abnormalities and limitations of mobility and pain degrade quality of life. In fact, standardized tests for quality of life in dystonia fall in the same range as patients with Parkinson's disease, multiple sclerosis, and stroke (8, 9).

## The Need for New Treatments

### Existing Treatments

Current treatments include physical therapy to address spasms or pain. Oral treatments are available to target the causal mechanisms for a few rare dystonia subtypes (13), but most are treated symptomatically with benzodiazepines, anticholinergics, or muscle relaxers (14, 15). The botulinum toxins are considered first-line treatments for many patients (16). Surgical interventions are offered for severe, medically refractory dystonias (17, 18). Deep brain stimulation (DBS) is most popular, but ablative procedures involving the basal ganglia or thalamus can also be helpful (19).

Despite these many treatment options, all have significant limitations. Physical therapy is popular; but benefits are variable and often short lived. There are many small open trials describing their value, but the largest and most rigorous studies fail to show any consistent benefits (20, 21). The most popular oral agents produce only partial benefits and doses are limited by side effects (3, 15).

The botulinum toxins suffer limitations too. Since they must be injected into affected muscles, they are most useful in the focal and segmental dystonias where a small number of muscles can be targeted. Because their benefits last only 2–4 months, injections must be repeated 3–4 times yearly. Despite dramatic efficacy on standardized tests of motor function in clinical trials, the botulinum toxins produce low levels of patient satisfaction, especially toward the end of a treatment cycle (22–24). Longitudinal studies have indicated that ~30% of patients discontinue using botulinum toxins (25), and cross-sectional studies indicate ~40% of patients are not using botulinum toxins (14). The reasons for low patient enthusiasm are only partly understood but appear to include lack of efficacy, side effects, difficulty in finding experienced injectors, hassle associated with repeated injections, and cost (24, 25).

For DBS, outcomes depend on etiology (e.g., genetic subtype or acquired) and certain clinical characteristics (e.g., age, duration, and combination with other problems) (17, 18, 26, 27). Therefore, DBS is not a suitable solution for many patients. Immediate complications are uncommon, but include 1–2% risk of stroke or infection. In addition, proper programming requires an experienced team, and it may take many months to optimize. Long-term complications are not uncommon, such as lead migration, equipment failure, or infection. In summary, all existing therapies provide at least partial relief of symptoms for many individuals with dystonia, but all have significant limitations.

### Experimental Therapeutics

When considering the development of novel therapeutics, clinical and etiological heterogeneity among the dystonias creates challenges. On the one hand, the different clinical manifestations seem to require different management strategies. In addition, the varied biological substrates may require targeting different mechanisms. On the other hand, several observations imply that certain forms of dystonia are mechanistically related (28, 29). In fact, there already are some treatments that have broad efficacy across many clinically and etiologically distinct subtypes, such as anticholinergics, botulinum toxins, and DBS.

These observations have encouraged attempts to identify the mechanisms that are shared across multiple types of dystonia. These mechanisms then become attractive targets for therapeutic interventions that may be useful across certain subgroups (30–32). At the genetic level, the identification of a large number of genes that may cause dystonia has facilitated the identification of several molecular mechanisms that are shared by at least certain subgroups of dystonia (5). For example, numerous studies in both animals and humans have linked dystonia with altered dopamine transmission. Although there are numerous reports describing good responses of certain cases to dopamine-related drugs, they are not generally effective treatments for most types of dystonia. It has been suggested that the failure of prior studies to demonstrate more consistent benefits might result from etiological heterogeneity, and clinical trials in more selected populations may be needed (33).

Pharmacological studies have also pointed to striatal cholinergic pathways as a common theme spanning several different types of dystonia in both animal models (34) and human studies (35). Although anticholinergic drugs can be at least partly effective across many different types of dystonia in humans, they are often poorly tolerated due to side effects including cognitive impairments, memory loss, dry mouth, blurred vision, constipation, and urinary retention. Current clinically available anticholinergics such as trihexyphenidyl non-specifically block muscarinic receptors. Numerous studies have focused on identifying novel compounds that may address these limitations (36). For example, by developing anticholinergics with more selective effects on the relevant muscarinic receptors in the striatum, it may be possible to avoid the many side effects that arise from non-specific blockade of receptors in the cortex or autonomic system.

Another common theme has involved abnormalities of neuronal excitability or neural plasticity among individuals with different types of dystonia (37). Glutamate receptors play a key role in neuronal excitability. Antagonists targeting several different subtypes of glutamate receptors (AMPA, NMDA, and mGluR5) have been shown to reduce dystonic movements or normalize abnormal striatal physiology in several animal models of dystonia (38). In humans with cervical dystonia, an open label study described small improvements with the non-selective glutamate antagonist riluzole (39), and there are anecdotal reports describing improvement with amantadine, a weak NMDA antagonist. These findings have led to interest into more methodical studies of repurposing glutamate-related drugs as potential therapeutics for dystonia. For example, the AMPA antagonist perampanel is FDA approved for epilepsy, and a trial for subjects with cervical dystonia has recently concluded recruitment (Clinicaltrials.org, NCT02131467).

Numerous other mechanisms are actively being studied as therapeutic targets for dystonia. At the end of year 2020, clinicaltrials.gov listed a total of 291 clinical studies for dystonia. Of this total, 156 have been completed and 49 are actively recruiting. Many of these are clinical trials of novel therapeutics (Table 1). However, many are small or unblinded pilot trials, and larger more rigorous trials are needed. Clinical trial readiness is therefore an immediate need. This readiness involves multiple ingredients including easy identification of research subjects for efficient recruitment, thorough understanding of phenotypic heterogeneity and diagnostic criteria for relevant subtypes of subjects, baseline information on how the disorder evolves over time, easy identification of experts who can participate in trials, clinimetrically sound measurement outcome tools for clinical trials (objective measurement tools and patient-reported outcomes), and fully objective or biomarker measures.

**Table 1:** Selected clinical trials on dystonias (https://clinicaltrials.gov).

**Drug/Intervention**	**Targeted mechanism**	**Type of dystonia**	**Study design**	**Date**
Ampicillin	Immune system	DYT1 dystonia	Phase I double-blind	2011–2017
DBS	Basal ganglia or thalamic nuclei	Dystonia and other disorders	Open label-single group assignment	2011–Present
Levetiracetam	Synaptic neurotransmission	Oromandibular and cranial dystonia	Phase II double-blind	2014–2017
Botulinum toxin plus physical therapy	Neuromuscular junction and musculoskeletal activity	Cervical dystonia	Phase 4 randomized	2014–2017
DBS	Pallidal and thalamic nuclei	Secondary hemi-dystonia	Phase I randomized	2015–Present
Hand physiotherapy	Individual finger movement training	Writer's cramp	Randomized-double blind	2016–Present
Perampanel	Glutamate receptor, AMPA	Cervical dystonia	Phase I/IIa open-label	2017–2020
Zolpidem	GABAa receptor chloride channel modulator/agonist	Writer's cramp or musician dystonia	Phase I crossover	2017–Present
Sodium oxybate	GABAb receptors	Laryngeal dystonia	Phase II/III double-blind	2017–Present
Daxibotulinumtoxin A	Neuromuscular junction	Cervical dystonia	Phase III double-blind	2018–2020
Daxibotulinumtoxin A	Neuromuscular junction	Cervical dystonia	Phase III open-label	2018–Present
Botulinum toxin and treadmill	Neuromuscular junction	Cervical dystonia and blepharospasm	Pilot monocentric, non-randomized, controlled	2019–Present
Deutetrabenazine	VMAT2 inhibitor	Dystonia	Phase I/II open-label	2020–Present
Tele-yoga	Mind-body awareness	Cervical dystonia	Single group intervention	2020–Present

*DBS, deep brain stimulation; GABA, gamma-amino butyric acid; VMAT2, vesicular monoamine transporter type 2*.

## The Dystonia Coalition (DC)

### Mission

The DC was established to address some of the challenges associated with research in rare disorders by facilitating large-scale collaborations. Its main focus has been on studies that address clinical trial readiness. The DC has focused its major projects on key unmet needs for translating scientific discoveries into potential new therapies. These unmet needs are identified via focused workshops, which are conducted in collaboration with Patient Advocacy Groups (PAGs). The main needs have included developing a better understanding of the phenotypic heterogeneity and evolution of various types of dystonia, more precise and widely accepted diagnostic criteria, appropriate measurement tools to monitor patients in clinical trials, and identification of useful biomarkers.

### Sponsorship and Endorsement

The DC is sponsored in large part by the National Institute of Neurological Disorders and Stroke (NINDS) and Office of Rare Diseases Research (ORDR) in the National Center for Advancing Translational Sciences (NCATS) at the National Institute of Health (NIH) through grants NS065701, TR001456, and NS116025. The DC is part of the NIH Rare Diseases Clinical Research Network (RDCRN), an initiative of the NIH to encourage collaborative research for all types of rare disorders (www.rarediseasesnetwork.org). The DC also receives critical support and sponsorship from PAGs, industry, professional societies, and relevant study groups.

PAGs have been integrally involved in all major activities of the DC. So far, the DC has engaged 17 PAGs across four countries in its different projects. Many of these regularly contribute to the DC mission (Table 2). PAGs have been integrally involved in identifying research topics and designing DC projects, developing a focus and plan for DC annual meetings, supporting studies of particular interest, supporting junior investigators, and facilitating patient recruitment. The Dystonia Medical Research Foundation (DMRF), in particular, plays an essential logistical role for the DC infrastructure, at no additional cost. In addition to aiding the organization of DC meetings and reviewing projects submitted for DC funding, DMRF staff provide support for managing subcontracts for paying all recruiting sites for the various DC projects. This unique model provides enormous savings for both research costs and time, enabling DC investigators to focus on clinical and scientific needs.

**Table 2:** Patient advocacy groups currently affiliated with the Dystonia Coalition.

**Patient Advocacy Group**	**Country**
Benign Essential Blepharospasm Foundation	USA
Cure Dystonia Now	USA
Dystonia Europe	Belgium
Dystonia Ireland	Ireland
Dystonia Medical Research Foundation	USA
Dystonia Medical Research Foundation, Canada	Canada
National Spasmodic Dysphonia Association	USA
National Spasmodic Torticollis Association	USA
The Dystonia Society	USA
Tyler's Hope	USA

### Coordination of Sites

**Figure 1:**
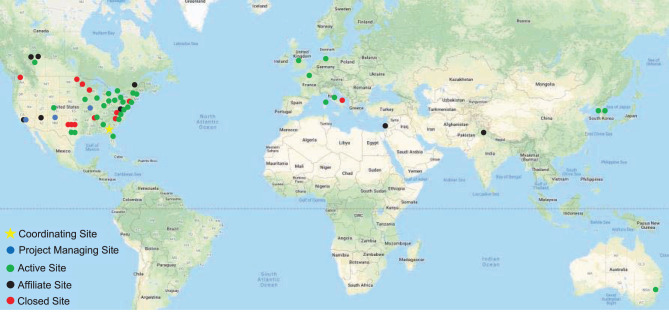
Dystonia Coalition sites. The main coordinating center is at Emory University (star). Sites responsible for directing large multicenter projects are shown in blue. Green shows sites that recruit patients for various studies or are recipients of Pilot Project grants or Career Awards. Affiliate sites are shown in black, and closed sites are shown in red.

The DC has had an open-door policy in which new investigators and institutions may join the effort at any time. The DC began in 2009 with eight sites but has since engaged 56 sites in the North America, Europe, Asia, and Australia (Figure 1, Supplementary Table 1). Many patients come to these centers for expert clinical care, as well as research opportunities. The DC sites are grouped in three tiers. As capabilities and interests change over time, centers may change tiers. *Affiliate Centers* are sites that may not have the ability to recruit subjects or direct projects but wish to remain informed about the DC activities and opportunities. *Recruiting Centers* are sites with sufficient expertise and clinical volumes to recruit subjects for clinical research projects. *Project Centers* are sites that take responsibility for directing multicenter clinical research projects. Individual investigators at these sites are given responsibility for developing and implementing projects using the DC infrastructure.

**Figure 2:**
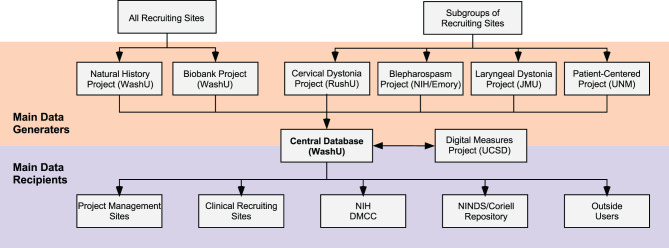
Data collection and sharing. Data for large multicenter projects are organized by individual sites (orange) and collected into a central database for checking, storage, and distribution. All Recruiting Sites (top left) may recruit subjects for the Natural History Project and the Biobank Project. Subgroups of Recruiting Sites are selected to participate in the other large multicenter projects including the Cervical Dystonia Rating Scale Project, The Blepharospasm Diagnosis and Rating Scale Project, the Laryngeal Dystonia Diagnosis and Rating Scale Project, and the Patient-Centered Outcomes Project. The Digital Measures Project analyzes video data collected by all projects. Data submitted to the central database are verified and organized and returned to the sites who manage the large multicenter projects, and are also shared with multiple additional users. For example, Recruiting Sites may request a summary of data they entered. Data are also shared with the National Institutes of Health (NIH) and the Data Management and Coordinating Center (DMCC) of the Rare Diseases Clinical Research Network. Subsets of data are also shared with the National Institute of Neurological Disorders and Stroke (NINDS) Repository at Coriell and with other users by request. DMRF, Dystonia Medial Research Foundation; JMU, James Madison University, RushU, Rush University; UCSD, University of California in San Diego; UNM, University of New Mexico; WashU; Washington University in St. Louis.

All DC activities are centrally coordinated. The coordinating center supervises the conduct and progress of its main clinical research projects, its smaller pilot projects, and its career awards. It also supervises the annual meeting and other activities. For multicenter projects, data are entered via the internet into a central database (Figure 2). Training webinars are held for recruiting sites. These webinars address protocol details such as recruitment goals, participant eligibility, inclusion/exclusion criteria, forms/questionnaires, data entry, and reimbursements. To ensure others outside the DC are aware of its activities, there are also annual meetings that describe all DC projects, accomplishments, how to get involved, or how to access data or materials. The annual meeting is not restricted to members of the DC; it is open to all academic investigators and their staff, PAG members, and representatives from NIH, and industry.

**Figure 3:**
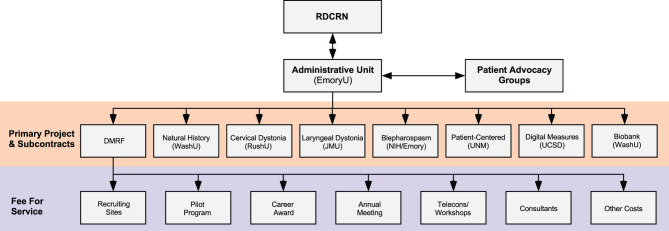
Financial structure. The majority of funding comes from the NIH Rare Diseases Clinical Research Network (RDCRN), although some funding and other resources also come from Patient Advocacy Groups. The Administrative Unit at Emory University provides payments by direct subcontracts to sites that organize large international multicenter projects. The Administrative Unit also has a subcontract with the Dystonia Medical Research Foundation (DMRF), which is responsible for disbursing funds on a fee-for-service basis to Recruiting Sites (depending on numbers of subjects recruited), smaller projects (such as Pilot Grant Projects or Career Awards), meetings, consultants, and others. JMU, James Madison University; NIH, National Institutes of Health USA; NINDS, National Institute of Neurological Disorders and Stroke; RushU, Rush University; UCSD, University of California in San Diego; UNM, University of New Mexico; WashU, Washington University in St. Louis.

The central coordinating center also manages the financial aspects of paying other centers for specific activities. It uses a direct subcontract to reimburse sites for the effort it takes to manage large clinical research projects (Figure 3). All other activities are financially managed through a subcontract with the DMRF. For example, *Recruiting Sites* are paid on a fee-for-service basis for each subject they recruit. The cost per subject depends on the study they were recruited for, and how much effort it takes the site to collect all data and samples for the study. Pilot projects and Career awards are also paid through the DMRF.

### Sharing Policies

The DC's open-door policy and broad collaborations have led to the collection of unprecedented amounts of detailed clinical data, video recorded examinations, and DNA samples from large numbers of dystonia subjects from different projects. Data and materials from DC projects are shared broadly with investigators both inside and outside the DC (Figure 2). All requests for data or material are granted, provided that the project has local IRB approval and does not directly conflict with ongoing DC studies. Access to DC data and materials is available via three different processes.

Data or materials may be requested directly from the DC through the *Data and Materials Request Form* (www.dystoniacoalition.org). Access to any original unpublished data or materials collected and stored by the DC is supervised by its Executive Committee. Data shared directly by the DC are provided in a de-identified manner, with a code number only. Video recordings of the face are classified as Protected Health Information (PHI) according to Health Insurance Portability and Accountability Act (HIPAA), and therefore are considered identifiable data. These recordings are shared only with extra security provisions. Data and materials collected by the DC also are compliant with the European General Data Protection Regulation (EU GDPR). DC policy requires all investigators requesting data or samples to sign a standard Bylaws agreement, which explicitly outlines the rights and responsibilities for sharing, as well as how investigators who contributed the material are most appropriately acknowledged.

Some of the de-identified key data elements collected by the DC are also sent to the NINDS Human Genetics Resource at Coriell, along with a blood sample for DNA extraction (www.coriell.org). Since the NINDS biorepository is a public resource, data and materials are collected by Coriell from non-DC members too. All materials are distributed by the NINDS Biorepository directly to qualified investigators by direct request.

De-identified data and materials are also stored by the NCATS-designated Data Management & Coordinating Center (DMCC). Sharing of these materials is governed by the policies and procedures of the RDCRN (www.rarediseasesnetwork.org/). Historically, materials stored by the DMCC have been subject to an embargo period during the period of active collection, analysis, and reporting by DC members.

Since 2009, the DC has received 47 requests for data or materials for studies that were beyond the scope of its existing projects. Except for projects that competed directly with ongoing projects, all requests were granted. Many of these projects have since been completed and published, or served as pilot data for grant proposals. Results from some of these projects are summarized in Tables 3–5.

**Table 3:** Dystonia Coalition career development award program recipients.

**Recipient**	**Institution**	**Year**	**Project title**
M. Carbon-Corell, Ph.D.	Feinstein Inst, Manhasset, NY, USA	2009	Sensorimotor network activity as a functional imaging marker for dystonia
M. Zurowski, MD	Univ Toronto, Canada	2010	Development of a psychiatric screening tool for cervical dystonia
A. Espay, MD	Univ Cincinnati, Cincinnati, OH, USA	2010	Sensory and emotional processing in psychogenic dystonia: a functional magnetic resonance imaging study
M. Karimi, MD	Washington Univ, St. Louis, MO, USA	2011	Basal ganglia induced plasticity in primary cervical dystonia
T. Kimberley, PT, Ph.D.	Univ Minnesota, Minneapolis, MN, USA	2011	Determining the efficacy of synergistic intervention in focal hand dystonia with repetitive transcranial magnetic stimulation and sensorimotor retraining
B. Berman MD	Univ Colorado, Denver, CO, USA	2012	Functional connectivity of the basal ganglia in primary focal dystonia: a pilot study
A. Wagle-Shukla, MD	Univ FL, Gainesville, FL, USA	2012	Subthalamic nucleus DBS in primary cervical dystonia: a pathophysiological insight
M. Bologna, MD	Univ Rome, Italy	2013	Effects of cerebellar theta-burst stimulation on arm and neck movement kinematics in patients with primary dystonia
S. Pirio-Richardson, MD	Univ New Mexico, Albuquerque, NM, USA	2013	Identification of optimal stimulation site for cervical dystonia symptoms: an exploratory study
D. Peterson, Ph.D.	Univ California, San Diego, CA, USA	2013	The contribution of blinks and spasms to blepharospasm severity
D. Arkadir, MD	Hadassah Univ, Israel	2014	Reinforcement learning in DYT1 dystonia
A. Shaikh, MD	Case Western, Cleveland, VA, USA	2016	Physiology of head tremor in cervical dystonia
K. Udupa, MD	Univ Toronto, Canada	2016	Phase-amplitude coupling of local field potentials in internal globus pallidus in dystonia
M. Hammer, MD	Univ Wisc, Madison, WI, USA	2017	Laryngeal somatosensory evoked cortical potentials in spasmodic dysphonia-an initial study to elucidate abnormal sensory mechanisms in laryngeal dystonia
N. Bukhari-Parlakturk, MD, Ph.D.	Duke Univ, Durham, NC, USA	2020	Non-invasive neuromodulation to study long-term plasticity mechanisms in task-specific dystonia
L. Rocchi, MD	Univ Rome, Italy	2020	Repetitive somatosensory stimulation in focal hand dystonia: a study on inhibitory circuitry plasticity of the somatosensory system and primary motor cortex

**Table 4:** Pilot projects funded by the Dystonia Coalition.

**Recipient**	**Institution**	**Year**	**Project title**	**Selected outcomes**
M. Ledoux, MD Ph.D.	Univ Tennessee, Memphis, TN, USA	2009	*THAP1* sequence variants in dystonia	(40, 41)
G. DeFazio, MD Ph.D.	Univ Bari, Italy	2010	Diagnostic guidelines and rating tools for blepharospasm	(42–44)
E. Roze, MD	University Hospitals Pitié Salpêtrière, Paris, France	2010	Cerebellar cortical plasticity in focal dystonia	(45)
K. Bhatia, MD FRCP	Univ Coll London, UK	2010	DYT6: A window to mechanisms in primary dystonia?	(46)
D. Peterson Ph.D.	Univ California, San Diego, CA, USA	2010	Increasing CERTainty in blepharospasm	(47)
C. Klein, MD	Univ Lubeck, Germany	2011	Endophenotypes in focal dystonias	In progress
H. Houlden, MRCP, Ph.D.	Univ College London, UK	2011	Neuropathology of DYT1 and DYT6 dystonia	(46)
S. Frucht, MD	Mt. Sinai School of Med, NY, USA	2013	Rating scales for musician's dystonias	(48)
S. Eichenseer, MD	Rush Univ, Chicago, IL, USA	2013	A novel method for rating scale assessment in cervical dystonia	Completed
M. LeDoux, MD Ph.D.	Univ Tennessee, Memphis, TN, USA	2013	Targeted sequencing in primary dystonias	(49)
H. Jinnah, MD Ph.D.	Emory Univ, Atlanta, GA, USA	2013	Resource for induced pluripotent stem cells	In progress
S. Norris, MD	Washington Univ, St. Louis, MO, USA	2013	Functional magnetic resonance imaging in laryngeal dystonia and muscle tension dysphonia	(50)
K. Lohmann, Ph.D.	Univ Luebeck, Germany	2013	Whole genome sequencing in focal dystonias	In progress
M. Zurowski, MD	Univ Toronto, Canada	2014	Development of a psychiatric screening tool for cervical dystonia	(51–53)
J. Mink, MD	Univ Rochester, NY, USA	2015	A rating scale for children with dystonia	Completed
M. Hammer, MA, Ph.D.	Univ Wisconsin, Whitewater, WI	2020	Non-invasive mechanosensory perturbation technique to test voice-related motor and somatosensory cortical responses in spasmodic dystonia	In progress

**Table 5:** Pilot projects supported with Dystonia Coalition data or materials.

**Recipient**	**Institution**	**Year of request**	**Topic**	**Status**
Neepa Patel, MD; J. Jankovic, MD	Baylor College of Medicine, Houston, TX, USA	2012	Sensory tricks in cervical dystonia	(54, 55)
M. Zaribaf; G. Kilic-Berkmen, Ph.D.	Emory Univ, Atlanta, GA, USA	2012, 2019	Family structures in dystonia	In progress
A. Shaikh, MD	Emory Univ, Atlanta, GA, USA	2013, 2016	Tremor in dystonia	(56–60)
T. Douglas, Ph.D.; G. Kilic-Berkmen, Ph.D.	Emory Univ, Atlanta, GA, USA	2013, 2019	Patterns of segmental and cervical dystonia	In progress
H. Sarva, MD; S. Bressman, MD	Mt Sinai, New York City, NY, USA	2014	Long term clinical outcomes in DYT1 dystonia	In progress
S. Norris, MD	Washington Univ, St. Louis, MO, USA	2014, 2016	Clinical characteristics of cervical dystonia	(61)
J. Junker, MD	Univ Lubeck, Germany	2015	Non-motor features of dystonia	(62)
J. Junker, MD; N. Bruggeman, MD	Univ Lubeck, Germany	2015-2017	Alcohol-responsiveness in Dystonia	(63)
J. Junker, MD	Univ Lubeck, Germany	2015, 2017	Quality of life in dystonia and its predictors	(9)
A. Shaikh, MD	Case Western Reserve Univ, Cleveland, OH, USA	2015	Quantitative analysis of dysphonia and voice tremor	In progress
S. Pirio-Richardson, MD	Univ New Mexico, Albuquerque, NM, USA	2015, 2017	Patterns of medication use	(14, 31)
L. Scorr, MD	Emory Univ, Atlanta, GA, USA	2016, 2020	Descriptive study of oromandibular dystonia	Manuscript submitted
Y. Sun, Ph.D.	Emory Univ, Atlanta, GA, USA	2016	Genome-wide association study for cervical dystonia	Manuscript in revision
Y. Sun, Ph.D.	Emory Univ, Atlanta, GA, USA	2016	Metabolomics in cervical dystonia	(64)
N. Patel, MD	Henry Ford Health System, Detroit, MI	2016	Substance abuse in dystonia	(65)
A. Espay, MD	Univ Cincinnati, Cincinnati, OH, USA	2016	Tremor in cervical dystonia	(57)
L. Froescheke, Ph.D.	Elmhurst College, Elmhurst, IL, USA	2016	Phonatory breaks in spasmodic dysphonia	(66)
A. Morris, MD	Washington Univ, St. Louis, MO, USA	2016–2018	Acoustic quantification of laryngeal dystonia	(67)
B. Berman, MD	Univ Colorado, Denver, CO, USA	2016, 2017	Psychiatric symptoms in dystonia	Merged with related project
C. Klein, MD Ph.D.	Univ Lubeck, Germany	2017	Penetrance and risk modifying variants in dystonia	In progress
D. Peterson, Ph.D.	UCSD and Salk, LA Jolla, CA, USA	2017	Objective phenotyping in cervical dystonia	In progress
H. Sarva, MD	Cornell, New York City, NY, USA	2017	Gait in blepharospasm	In progress
V. Fung, MD; F. Chang, MD	Westmead Hospital, Australia	2017	Torticollis in hemidystonia	In progress
S. Cho, MD; M. Hallett, MD	NINDS, Bethesda, MD, USA	2017	Sensory tricks in blepharospasm	Merged with related project
B. Berman, MD	Univ Colorado, Denver, CO, USA	2017	Patterns of spread in dystonia	(68)
S. Norris, MD	Washington Univ, St. Louis, MO, USA	2018, 2020	Spread of limb dystonia	(12)
D. Martino, MD	Univ Calgary, Canada	2018	Demographic and clinical predictors of spread in adult-onset idiopathic dystonia	In progress
M. Powell, Ph.D.	Vanderbilt Univ, Nashville, TN, USA	2019	Artificial intelligence for diagnosing, and monitoring laryngeal dystonias	In progress
C. Klein, MD Ph.D.	Univ Lubeck, Germany	2019	Genome-wide association study for dystonia	In progress
N. Harrison, MD; S. Norris, MD	Washington Univ, St. Louis, MO, USA	2019	Shoulder dystonia in upper extremity vs. cervical dystonia	In progress
A. Cotton; H. A. Jinnah, MD Ph.D.	Emory Univ, Atlanta, GA, USA	2019	Patient-reported outcomes vs. clinical rating scales	In progress
E. Reid, Ph.D.	Loma Linda Univ, Loma Linda, CA	2019	Phonetic analysis of spasmodic dysphonia	In progress
K. Lohmann, MD	Univ Lubeck, Germany	2020	Large-scale sequencing of dystonia	In progress
K. Peall, MD	Cardiff Univ, United Kingdom	2020	Predictive models for phenotypic subgroups across the dystonias	Manuscript submitted
L. Scorr, MD	Emory Univ, Atlanta, GA, USA	2020	A descriptive study of blepharospasm	In progress
M. Sousa, MD; S. Fox, MD	Univ Toronto, Canada	2020	Anxiety in cervical dystonia	In progress
M. Tosin, Ph.D. student	Rush Univ, Chicago, IL, USA	2020	Head tremor in cervical dystonia	In progress
N. Koirala, Ph.D.	Haskins Lab, New Haven, CT, USA	2020	Machine learning algorithms for detection of dystonia	In Progress

### Co-authorship Policies

In a large collaborate effort, it is important to appropriately acknowledge the varied effort of the many different individuals involved. Guidelines for these acknowledgments are shared with all DC members in a written document that all investigators sign. In brief, the effort for recruiting patients and conducting study procedures is acknowledged in part by including recruiting investigators on relevant publications. The investigators conducting the study may offer authorship to any relevant study team members. In addition, other investigators who recruited patients essential to the study may also be offered co-authorship. Typical criteria for authorship for recruiting investigators include at least 20 subjects for the study under consideration, and evidence for ongoing and active participation as judged by the recruitment of at least one subject per month. This policy discourages investigators from assuming they will be co-authors for recruiting only a few cases, or from recruiting 20 cases and expecting co-authorship for all future studies. The study organizers notify the site PI of any publication taking advantage of cases they recruited, and the site PI is asked to nominate the most appropriate co-author at the site. If more than 40 cases were recruited, the site PI can nominate two co-authors, and an additional co-author for every additional 20 cases recruited. Authorship must also meet the usual criteria outlined by Council of Science Editors. All other active investigators are listed in acknowledgments.

## International Multicenter Dystonia Coalition Projects

### Natural History Project

Historically, there has been a relatively limited appreciation of the full phenotypic spectrum and evolution of all types of dystonias. Most evidence came from relatively small studies, often focusing on a single subtype of dystonia. Most studies came from single centers, leading to differences of expert opinion.

A thorough understanding of clinical features and especially their evolution with time is an essential prerequisite for testing any disease-modifying therapies that could halt or slow progression. The aim of *Natural History Project* has been to better characterize the heterogeneity of clinical manifestations in dystonia and how these manifestations evolve over time. Centers in multiple countries collect a standardized dataset that they enter into a central database, and record a standardized video shown in Supplementary Table 2 (69). All data and videos are checked for accuracy and completeness. Since 2009, more than 3,200 cases have been recruited, many of whom continue to be followed. This study has led to several comprehensive articles that have raised awareness of the phenotypic spectrum of dystonia (49, 56–58, 62, 63, 70). This study has also led to several multicenter articles demonstrating progression of adult-onset dystonias over time (12, 61, 68). These studies provide critical baseline information for testing any future disease-modifying treatments, by revealing how many patients would have to be studied, and for how long (68). This project also led to several articles summarizing evidence that available treatments are not as satisfactory as commonly believed (25, 71, 72).

Another outcome from this project relates to the very definition of dystonia. Prior to starting of DC, the definition of dystonia varied in different parts of the world. Furthermore, many subtypes were recognized, but they were organized in different ways. This heterogeneity led to confusion in the interpretation of many studies because of diagnostic uncertainties of the patient cohorts studied. The DC sponsored a series of meetings with PAGs in America and Europe to develop an internationally accepted consensus on its definition (1, 73). The same group also presented a new classification for the many subtypes. The results of the consensus group were published in 2013, they were accepted internationally almost immediately, and the article has been cited more than 1,287 times already. Now, when articles on dystonia are published, most investigators understand exactly what subgroups are being studied.

### Clinical Rating Tools for Cervical Dystonia

All clinical trials need good outcome measures. The main goal of this project was to revise and re-evaluate the most popular clinical rating scale for cervical dystonia, the TWSTRS. This scale had known deficiencies in its clinimetric properties including inconsistent scoring among items, double weighting of duration factors, and variable approach to different aspects of the disorder (74, 75). Additionally, the scale neglected non-motor features such as depression and anxiety, which are known to have a strong impact on quality of life (9, 51, 76–82). This project was designed to address these shortcomings by producing and clinimetrically validating the Comprehensive Cervical Dystonia Rating Scale, which has three modules addressing motor features, non-motor features, and quality of life. This project completed recruitment in 2014, with 209 subjects recruited from 10 sites (52, 53). Thus, a tangible deliverable from this project is a fully validated and comprehensive rating scale for both motor and non-motor features of cervical dystonia that can be used in modular format or in whole.

Although the primary goal of this project has been completed, the rich database collected inspired a number of secondary studies (49, 54, 61, 83, 84). Ongoing work involves testing the new scale by experts in other countries for international validation, and development of a teaching tape for its use. Most importantly, this project has served as a model for other subtypes of dystonia, where clinical rating scales were less well-developed or absent.

### Diagnostic Tools for Laryngeal Dystonia (Spasmodic Dysphonia)

Workshops aimed at delineating research priorities for laryngeal dystonia sponsored by the National Spasmodic Dysphonia Association (NSDA) have repeatedly identified the lack of widely accepted diagnostic criteria and rating tools as major obstacles for clinical and basic research for laryngeal dystonias (85, 86). The goals of this project were parallel to those of the cervical dystonia project described above. However, in the case of laryngeal dystonia, widely accepted rating tools were not available. This project completed recruitment goals in 2015 with 197 subjects that had a detailed evaluation by a multidisciplinary team that included a laryngologist, neurologist, and speech language pathologist. The evaluation included audio and video recordings of voice characteristics during standard voice tasks, audiovisual recordings of laryngoscopy to evaluate the vocal folds with standard tasks, audiovisual recordings of a standard neurological exam, and a blood sample for the DNA biorepository. Initial analyses revealed strikingly poor diagnostic agreement, even among the most experienced experts. As a result, rating tools could not be developed. Instead, a Delphi panel was established to develop more universally acceptable diagnostic criteria (70). Thus, a tangible deliverable of this project is novel diagnostic criteria that may now be used to distinguish subtypes of laryngeal dystonia and to discriminate them from related voice disorders.

This project also led to numerous unexpected directions. For example, several investigators have accessed audiovisual recordings for different types of perceptual or acoustic analyses, including machine learning approaches, which are ongoing.

### Diagnostic and Rating Tools for Blepharospasm

Historically, there have been no widely accepted diagnostic criteria for blepharospasm and related craniofacial dystonias. Clinical rating tools were available, but suffered numerous limitations (75). Thus, the goal of this project was to address these needs. Diagnostic criteria and a novel clinical rating scale were first established in pilot studies (42, 43), and then tested in a larger international multicenter design. Eleven centers in four countries recruited 200 individuals with blepharospasm along with individuals with other disorders often mistaken for blepharospasm, such as tics or ptosis. Analyses of these data are nearly complete, and tangible deliverables from this project will be internationally validated diagnostic criteria and clinical rating scale that can support clinical trials.

### Patient-Centered Outcomes Project

The projects described above focus mostly on clinician-determined assessments. Sometimes, clinician assessments do not match patient views. For example, the botulinum toxins produce highly significant effects using clinician-rated scales for many types of dystonia, yet patients often report low levels of satisfaction (22, 23, 25, 71, 72, 87), with at least 30% discontinuing use (25). There are many reasons for frequent discontinuation of botulinum toxins, one of which has been dubbed the yo-yo effect (31). Typically, injections are required about every 3 months. Therapeutic benefits emerge within the 1st week and then wear off after 8–16 weeks, creating a cyclical response known as the “yo-yo” effect. Although this cyclical effect is widely known, there are few data describing its frequency, magnitude, and temporal aspects. In order to design clinical trials for any potential add-on therapy, it is essential to have clear understanding of the cyclical responses to botulinum toxins.

The aim of the *Patient-Centered Outcomes Project* is to delineate both between-subject and within-subject variations over time in response to the standard of care treatment with botulinum toxin, from the perspective of the patient. Existing tools to measure efficacy rely on clinical rating scales which are subjective, cumbersome for repeated frequent use, and require extensive expertise to apply. This project aims instead to develop a patient-facing tool on a hand-held electronic device, such as a smartphone. It will focus on the most common dystonias, cervical dystonia, blepharospasm, and laryngeal dystonia. This tool will have 10–15 disorder-specific questions that can be answered on a more frequent basis than existing scales (e.g., weekly), to provide a more direct and more precise temporal appreciation of responses over time. This tool will be ideal for any novel clinical trial that proposes an “add-on” therapy, as well as for comparing durations of responses among different botulinum toxins.

### Objective Measures Project

Current tools for diagnosis and assessment of severity depend almost entirely on subjective clinician-rated or patient-rated tools, but advances in modern technology have opened the door to more objective strategies. The *Objective Measures Project* aims to exploit technological advances in digital tools to measure the severity of dystonia. Specifically, this project will exploit advances in computer vision and machine learning to semi-automatically analyze common abnormalities evident in video recordings of blepharospasm, cervical dystonia, and laryngeal dystonia. This new technology could ultimately replace subjective clinical rating scales as outcome measures and enable remote assessment for telemedicine.

In pilot studies, this strategy was used to quantify blinks and spasms among subjects with blepharospasm (47). The results demonstrated good correlations with clinical rating scales. Additional studies will address other manifestations of blepharospasm, such as apraxia of eyelid opening. They will also exploit similar technology for assessment of abnormal head movements in cervical dystonia and abnormal vocal fold movements in laryngeal dystonia. Thus, an important deliverable from this project is a truly objective measure of abnormal movements in dystonia, which may be applied to videos for remote assessments.

### Biobank Project

Biomarkers can also provide valuable tools for clinical trials. Genes can provide useful diagnostic tools. However, existing genes account for only a small fraction of all subjects with dystonia and do not predict penetrance, severity, onset, or rate of progression. There are no practical biomarkers for addressing severity of the dystonias. Neuroimaging abnormalities provide a potential “endo-phenotype” (88, 89), but most are not practical as clinical biomarkers. Several studies also have identified subclinical defects in sensory function (90–92), but their significance and whether they can serve as biomarkers remains unclear.

The aim of the *Biobank Project* is to develop a resource that permits sharing of DNA samples with carefully annotated clinical data. This resource was started with DNA collection in 2009 and currently has more than 3,000 samples. This is the largest and most carefully clinically annotated biobank in the world. DNA samples have been accessed numerous times, for example, for validation studies (49). They have also been accessed for genome-wide association studies (GWAS) and whole exome sequencing (WES) studies, which are ongoing. In 2020, the Biobank added collection of RNA and plasma that will allow for additional studies including transcriptomics, proteomics or lipidomics, epigenomics, and others. A pilot study of metabolomics has provided hints that this approach may be successful (64). The goal is to create a resource for biomarker discovery and validation.

## Other Dystonia Coalition Activities

In addition to the large multicenter studies described above, the DC also encourages the development of new investigators and new studies relevant to dystonia. Like the large multicenter projects, these other activities focus on clinical or translational research. A collaborative approach is encouraged. Scientific advisory board members from dystonia PAGs are integrally involved in the review of potential new projects, results of the review process are shared with PAG leaders, and PAGs are involved in final project selection and often funding too.

### Career Development Award

The goal of the Career Development Award is to promote career development for investigators interested in research in dystonia and related rare disorders. The DC is particularly interested in applications aiming to exploit data and/or resources already collected by the DC or projects that encourage collaborations by involving different centers of the DC. All applicants may apply regardless whether they are part of the DC or not. US citizenship and affiliation with a US institution are not required. Most awards are directed toward junior faculty interested in developing careers in clinical and translational research in dystonia, but more senior investigators may apply if they are redirecting their efforts from another area of research to dystonia. Advanced postdoctoral fellows who are transitioning to their first faculty appointment may also be considered. Applications are reviewed by the DC, and successful applicants are asked to provide written progress reports. Since 2009, the DC received a total of 40 applications for this award and provided funding for 16 candidates in 4 different countries. A summary of recipients and their projects is provided in Table 3. Further information regarding this opportunity can be found at www.dystoniacoalition.org.

### Pilot Projects Program

The goal of the Pilot Projects Program is to foster promising pilot studies to a point where they can be published or compete for independent funding. The DC is particularly interested in applications focusing on clinical or translational projects with direct relevance to dystonia, projects aiming to exploit data and/or resources already collected by the DC, and/or projects that encourage collaborations by involving different centers of the DC. Applicants may come from DC centers, although membership in the DC is not required. US citizenship and affiliation with a US institution are not required. Applications are reviewed by the DC, and successful applicants are asked to provide written progress reports. Since 2009, the DC received 80 applications and provided funding for 16 applications in five different countries (Table 4). Most projects have received $10,000–50,000 in financial support. Further information regarding this opportunity can be found at www.dystoniacoalition.org.

The DC also supports Pilot Projects by providing DC data and materials, rather than direct financial support. For example, the DC has received more than 47 formal requests for data or materials. All requests were approved except for two, which were requests that overlapped with existing projects (Table 5). Further information regarding how to make a request for data or materials is described above in *Sharing Policies*, and at www.dystoniacoalition.org.

## Summary and Future Directions

The dystonias are a rare and very heterogeneous group of disorders. They have a profound impact on quality of life, and existing treatments all have significant limitations. New or improved treatments are sorely needed. There are multiple ongoing efforts to improve existing therapies or develop entirely novel approaches. Any novel approach to therapy will require rigorous clinical trials. As a result, the majority of studies by the DC have focused on clinical trial readiness. The DC has addressed the need to identify experts who can participate in trials. It has also conducted multiple studies of clinical heterogeneity among different dystonias, the progressive nature of some dystonias, diagnostic criteria, clinical rating tools, patient-reported outcomes, digital measurement tools, and biomarkers for diagnosis or severity. Along the way, the DC has supported more than 150 articles, numerous grant proposals, and 13 meetings or workshops.

The dystonia community looks forward to a day when all affected individuals can get a rapid and expert diagnosis, and ready access to effective treatments that control the debilitating consequences of the disorder. The dystonia community also looks forward to reaching a better understanding of the etiology and pathogenesis of dystonia, so that truly disease-modifying therapies can be designed to halt progress or even reverse it. For a rare disorder like dystonia, The Dystonia Coalition has demonstrated that broad collaborations and cooperation are essential to these goals.

## Author Contributions

GK-B and HAJ drafted the initial manuscript. JSP, CCo, MH, SPR, DAP, CCr, LJW, CL, and JT: reviewed and revised the manuscript. All authors contributed to the article and approved the submitted version.

## Conflict of Interest

The authors declare that the research was conducted in the absence of any commercial or financial relationships that could be construed as a potential conflict of interest.
