# Supplementary material for: The Dystonia Coalition: A Multicenter Network for Clinical and Translational Studies
Source: Front Neurol. 2021 Apr 8;12:660909. doi: 10.3389/fneur.2021.660909 (PMC8060489; doi:10.3389/fneur.2021.660909)
Supplement: Supplementary file 1 [file Table_1.pdf]

Supplementary 1. Dystonia Coalition Sites

NO	NAME OF SITE	PROPOSED STATUS
1	**Asan Medical Center, Seoul, South Korea**	Recruiting Site
2	Baylor College of Medicine, Houston, TX	Recruiting Site
3	Beth Israel Deaconess Medical Center, Boston, MA	Recruiting Site
4	Beth Israel Medical Center (Mount Sinai), New York, NY	Recruiting Site
5	Booth Gardner Parkinson's Care Center, Kirkland, WA	Closed
6	Case Western Reserve Univ, Cleveland, OH	Recruiting Site
7	**Children's Hospital Univ Medical Center, Montreal, Canada**	Affiliate Site
8	Duke University Medical Center, Durham, NC	Recruiting Site
9	Emory Univ, Atlanta, GA	Organizing Center<br>Recruiting Site
10	Feinstein Inst, NY	Closed-PI moved
11	Hadassah U, Israel	Affiliate Site
12	**Govind Ballabh Pant Institute, Delhi, India**	Potential Recruiting Site
13	Henry Ford Health System, Detroit, MI	Recruiting Site
14	James Madison Univ, VA	Closed-PI retired
15	Johns Hopkins Univ, Baltimore, MD	Recruiting Site
16	Lahey Clinic, Burlington, MA	Recruiting Site
17	Mayo Clinic, Phoenix, AZ	Affiliate Site
18	Medical College of Wisconsin, Madison, WI	Closed-PI moved
19	Methodist Hospital Research Institute, Houston, TX	Recruiting Site
20	Mount Sinai Medical Center, New York, NY	Closed-PI moved
21	National Institute of Neurological Disorders and Stroke, Bethesda, MD	Affiliate Site
22	Parkinson's Movements Disorders Center of Maryland, Elkridge, MD	Recruiting Site
23	Parkinson's & Movements Disorders Institute (PMDI), Fountain Valley, CA	Affiliate Site
24	Rush Univ, Chicago, IL	Recruiting Site
25	**Salpetriere Hospital, Paris, France**	Recruiting Site
26	Sanford Health, Fargo, ND	Closed-PI moved
27	**Seoul National Univ Hospital, Seoul, South Korea**	Recruiting Site
28	**Toronto Western Hospital, Toronto, Canada**	Recruiting Site
29	**Univ College London, London, UK**	Recruiting Site
30	Univ Alabama, Birmingham, AB	Recruiting Site
31	**Univ Alberta, Edmonton, Canada**	Affiliate Site
32	**Univ Bari, Bari, Italy**	Closed-PI moved
33	**Univ Cagliari, Cagliari, Italy**	Recruiting Site
34	**Univ Calgary, Alberta, Canada**	Recruiting Site
35	Univ Chicago, Chicago, IL	Affiliate Site

36	Univ Cincinnati, Cincinnati, OH	Recruiting Site
37	Univ Colorado, Denver, CO	Recruiting Site
38	Univ Florida, Gainesville, FL	Recruiting Site
39	Univ Iowa, Iowa City, IA	Recruiting Site
40	**Univ Luebeck, Luebeck, Germany**	Recruiting Site
41	Univ Maryland, Baltimore, MD	Recruiting Site
42	U Minnesota	Closed-PI moved
43	Univ New Mexico, Albuquerque NM	Project Site
		Recruiting Site
44	Univ Pennsylvania, Philadelphia, PA	Recruiting Site
45	Univ Rochester, Rochester, NY	Recruiting Site
46	**Univ Rome, Rome, Italy**	Recruiting Site
47	Univ Tennessee, Memphis, TN	Closed-PI moved
48	Univ Texas, Houston, TX	Closed-PI moved
49	Univ CA San Diego, San Diego, CA	Project Site
50	Univ Texas Southwestern, Dallas, TX	Closed-PI moved
51	U Wisconsin, Madison	Affiliate Site
52	Veracity Neuroscience LLC, Memphis TN	Recruiting Site
53	Virginia Commonwealth Univ, Richmond, VA	Recruiting Site
54	Wake Forest Univ, Winston Salem, NC	Closed-PI moved
55	Washington Univ in St. Louis, St. Louis, MO	Project Site
		Recruiting Site
56	**Westmead Hospital, Sydney, Australia**	Recruiting Site
