# Supplementary material for: The Dystonia Coalition: A Multicenter Network for Clinical and Translational Studies
Source: Front Neurol. 2021 Apr 8;12:660909. doi: 10.3389/fneur.2021.660909 (PMC8060489; doi:10.3389/fneur.2021.660909)
Supplement: Supplementary file 2 [file Table_2.pdf]

Supplementary 2. Dystonia Coalition Video Exam Protocol-V40

General Instructions

- Remove shoes and socks. Roll long pants up to make feet visible. Remove glasses. Remove food or chewing gum from mouth. Pull long hair away from neck in a clip or rubberband.
- Use a tripod. Ensure adequate lighting, especially for face. Prevent shadows by avoiding overhead or back lighting.
- Video should be taken from directly in front of the participant, torso square to camera, except where stated otherwise.
- Avoid recording the participant's name, date of birth, other identifiers, and other people.
- Each part should be recorded as a separate clip, then put together prior to upload to avoid unnecessary footage. Please also record the instructions given to the subject for each step.
- Record the following statement at start of video: **This is subject Dys#X** (*state age of subject if study ID is not available)* **enrolled at (site name) on (current date & year).**"
- Direct participant to rest hands flat in their lap and feet flat on the floor unless otherwise directed.

Part I: Participant sits in a chair without head support. Zoom camera in to capture all of head and shoulders only. Include whole head. Do not leave a lot of blank space above the head.

1. At rest, eyes open, **1 minute**
2. At rest, eyes closed gently, 10 seconds
3. Squeeze eyes forcibly closed, 5 times for 1 second each, then open eyes and observe for 10 seconds
4. Repeat each sentence or sound below, one at a time:
   a. "We mow our lawn all year" b. "We eat eggs every day" c. "He had half a head of hair"
   d. "The puppy bit the tape" e. "TaTaTa" f. "GaGaGa" g. "PaPaPa"
5. Hold long vowel sounds: "AHHHHHH" (5 seconds); then "EEEEEEE" (5 seconds)
6. Stick tongue out as far as possible and hold for 5 seconds
7. Open and close mouth as wide as possible, 2 times
8. Ask: "do you have trouble swallowing or choking?" If yes, ask: "occasional or frequent?"

Part II: Participant remains seated. Zoom camera out to capture upper body, including head and both upper limbs. Include whole head. Do not leave a lot of blank space above the head.

9. At rest, eyes **closed**: instruct participant to let head drift to its most comfortable (dystonic) position, 10 seconds
10. At rest, eyes **open**: assist subject verbally to reach a completely straight position of the head. Instruct participant to hold head in this position for 1 minute. If head drifts, do not ask participant to reposition.
11. Demonstrate **maximum range of head motion** by asking subject to move head **as far as possible**, each direction:
    a. Frontal view: turn head to right, hold 5 seconds. Then turn head to left, hold 5 seconds
    b. Frontal view: Tilt ear to right shoulder, hold 5 seconds. Tilt head to left shoulder, hold 5 seconds
    c. Lateral view (choose one side): Chin up, hold 5 seconds. Chin down, hold 5 seconds

Part III: Participant remains seated. Roll sleeves up for both arms. Participant may wear glasses if needed. Use the video exam worksheet on a stable writing table. Non-active hand should rest comfortably on table without holding paper. Do not allow any tricks or compensations. Zoom camera out to capture only upper body, including whole head and both upper limbs.

12. Write "TODAY IS A NICE DAY" 3 times with dominant hand, then with other hand
13. Draw spiral with dominant hand, then with other hand. Do not let drawing hand rest on paper or table
14. Hold tip of pen as close as possible to dot without touching paper for 10 seconds with dominant hand, then other hand
15. Examiner holds up written page so that it is clearly visible on the video
16. Extend arms/hands directly towards camera, palms up, hold 5 seconds; then palms down, hold 5 seconds
17. Flex elbows and hold hands/arms steady, palms down, without touching in front of chest, hold 5 seconds
18. Finger-to-nose test, far enough to make subject reach, slow enough to capture accuracy, 5 trials for each hand
19. Finger tapping (thumb and forefinger) 10 times for each hand, as big and fast as possible
20. Open and close hands rapidly and fully, 5 times (both hands simultaneously)

Part IV: Zoom camera out to capture entire body, including head and all limbs

21. Remain seated. Tap heel then toe on floor in rapid alternations, 5 repeated pairs each side
22. Standing frontal view, 5 seconds. Standing lateral view, 5 seconds. Standing back view, 5 seconds
23. Walk at least 10 steps away from camera (posterior view) and at least 10 steps towards camera (frontal view)
24. Walk on toes at least 10 steps away from camera, and on heels at least 10 steps towards camera
25. Walk in toe-heel-tandem, at least 10 steps away from camera and at least 10 steps back towards camera

Part V: Participant standing or sitting depending on task. Zoom as appropriate

26. Ask if there is anything that can be done to reduce the severity of the movement disorder (sensory trick). If yes, ask subject to demonstrate trick, and video at least 5 seconds to demonstrate the effect of trying the trick.
27. If participant has a task specific dystonia not captured in protocol above, such as playing musical instrument or chewing, please video the dystonic symptoms while participant performs the problem task.
